# Massive congenital cervicofacial desmoid-type fibromatosis in a 5-month-old infant

**DOI:** 10.1093/jscr/rjab206

**Published:** 2021-05-27

**Authors:** Mohammed S Albokashy, Mohammed S Halawani, Anoof T Eshky, Khalid Alsaad, Hatim A Khoja, Samir M Bawazir

**Affiliations:** Pediatric Division, Department of Otolaryngology/Head and Neck Surgery, Prince Sultan Military Medical City, Riyadh, Saudi Arabia; Pediatric Division, Department of Otolaryngology/Head and Neck Surgery, Prince Sultan Military Medical City, Riyadh, Saudi Arabia; Pediatric Division, Department of Otolaryngology/Head and Neck Surgery, Prince Sultan Military Medical City, Riyadh, Saudi Arabia; Department of Oral and Maxillofacial Surgery, Prince Sultan Military Medical City, Riyadh, Saudi Arabia; Department of Pathology and Laboratory Medicine, King Faisal Specialist Hospital and Research Center, Riyadh, Saudi Arabia; Pediatric Division, Department of Otolaryngology/Head and Neck Surgery, Prince Sultan Military Medical City, Riyadh, Saudi Arabia

## Abstract

Desmoid-type fibromatosis (DF) is a borderline tumor of soft tissues that has low malignant potential but described as infiltrative, locally aggressive and rapidly growing. In the pediatric population, it occurs in the head and neck. Presentation varies based on tumor size and location. Despite the high recurrence rate, surgical excision remains the modality of choice with. Here, we report a case of a 5-month-old boy, with extensive head and neck DF that was managed twice with conservative debulking surgery through a combined transoral-transcervical approach. On 2-year follow-up, he was gaining weight with no developmental delay and had no clinical evidence tumor regrowth.

## INTRODUCTION

The WHO classification of head and neck tumors, define desmoid-type fibromatosis (DF) as a borderline tumor of the soft tissues that has low malignant potential. DF is characterized by local aggressiveness with an ~20% local recurrence rate, but without metastasis [1]. The annual incidence of DF is presumed to be 2–4 per 1 million [2]. In the head and neck, it accounts for 12–15% of all desmoid tumors throughout the body with 25% of all desmoid tumors occur in children under 15 years of age [3]. The pathogenesis of desmoid tumors remains unclear. Proposed theories include: a genetic defect in the regulation of connective tissue growth and trauma. Associations with Gardner syndrome and familial adenomatous polyposis were also found with a 13% increase in prevalence [2, 3]. In the head and neck, vital structures including cranial nerves and great vessels make the local excision challenging, especially in children [4].

## CASE REPORT

**Figure 1:**
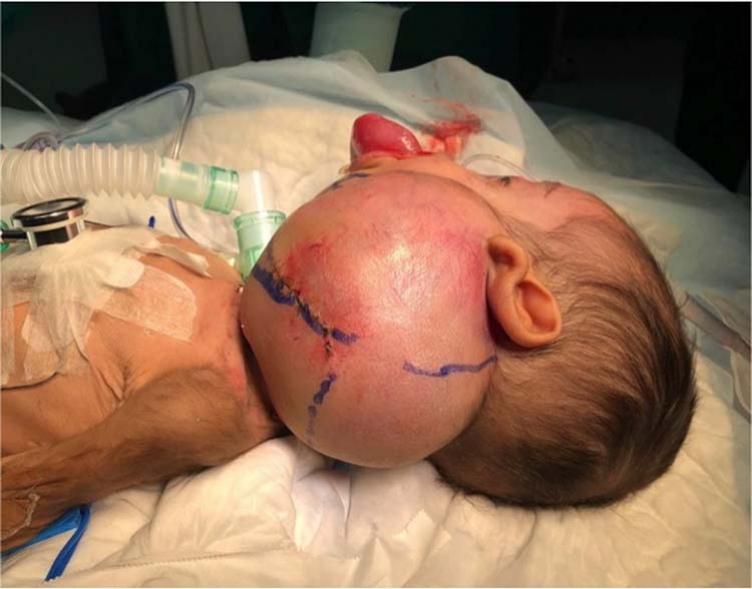
Presentation of the infant to our center.

**Figure 2:**
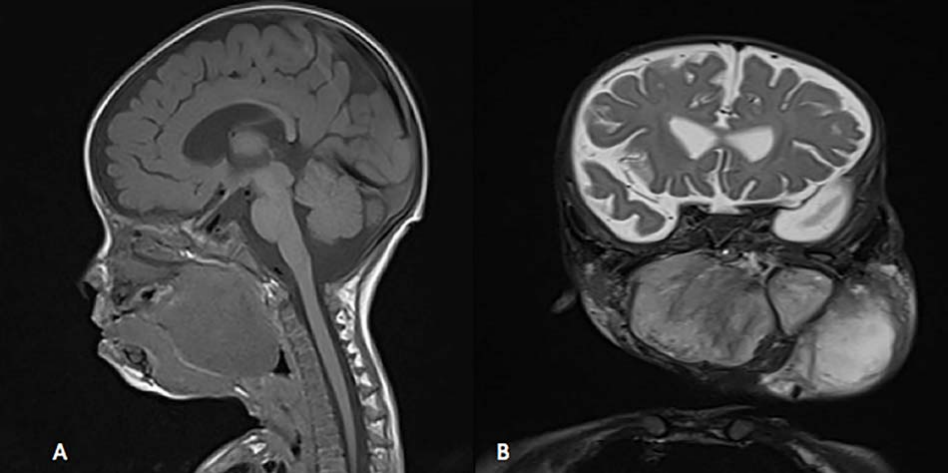
MRI, (**A**) sagittal view, showing the mass with extension into pharynx and oral cavity pushing the tongue base, (**B**) coronal view, the mass involving the neck, parapharyngeal space and pharynx up to base of skull.

**Figure 3:**
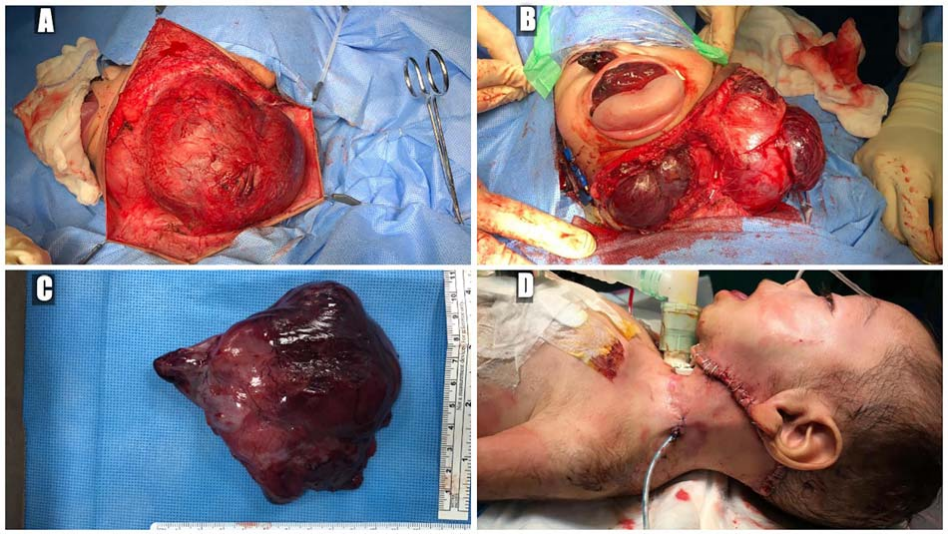
(**A**) trans-cervical approach, (**B**) trans-oral/trans-cervical excision of the mass, (**C**) post-excision size (12 × 12 cm) and (**D**) the patient post-operatively.

A 5-month-old boy was referred to our center. He presented with an early tracheostomy due to the presence of a left large congenital neck mass involving a large portion of the cervicofacial region. It was rapidly growing causing feeding and breathing difficulties. Upon examination, the mass was soft, mobile, non-tender and measuring ~10 × 6 cm on the left side of the neck extending from the mandible down to the clavicle. The mass was fungating from the nostrils and causing the tongue to protrude externally from the oral cavity (Fig. 1). A trans-nasal flexible laryngoscope revealed extension into the nasopharynx and oropharynx obscuring the airway. Magnetic Resonance Imaging showed mass enhancement with extension medially into parapharyngeal space, nasopharynx, oropharynx and superiorly to the skull base (Fig. 2). Incisional biopsy was performed which confirmed the diagnosis of a DF. A multidisciplinary team was formed and decision made to surgically excise the mass. Starting with a transcervical approach, the mass was dissected laterally from the great vessels, superiorly from skull base with some residual. Then, surgical debulking of the oral mass done by incision on the floor of mouth. Subsequently, the mass was directed from the nasopharynx, oropharynx and oral cavity through the floor of the mouth finally into neck incision. Post-operatively, the patient was in stable condition and was under intensive care observation (Fig. 3A–D).

**Figure 4:**
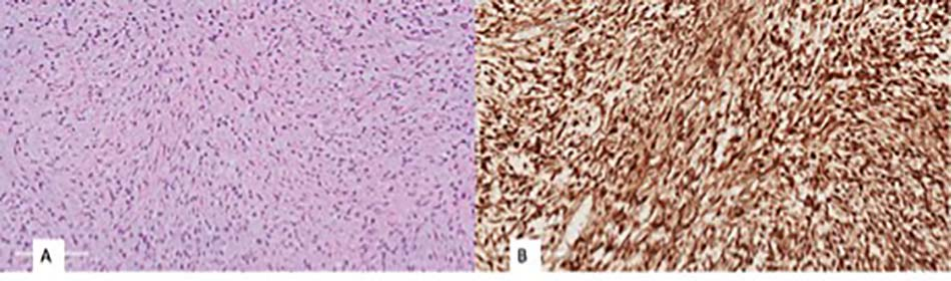
(**A**) monomorphic spindle cell fibroblastic proliferation with myxoid background (hematoxylin and eosin stain (20×)), (**B**) diffuse nuclear staining of B-Catenin.

Histopathological examination revealed a spindle cell neoplasm with long sweeping fascicles, myxoid/collagenized background (Fig. 4A). Tumor cells showed diffuse and strong nuclear staining for B-Catenin (Fig. 4B). After 6 months, the patient had developed a recurrence in the same site. He was then taken again for debulking. In 2-year regular follow-up, he was gaining weight with no developmental delay and he had no clinical evidence tumor regrowth.

## DISCUSSION

Aggressive infantile fibromatosis is the juvenile form of the musculoaponeurotic fibromatosis of adulthood. Fibromatosis is known to infiltrate and replace surrounding structures [5]. They are mesenchymal in origin and of two types: diffuse and desmoid [6]. Fibromatosis accounts for 0.03% of all neoplasms and 3% of all soft tissue neoplasms [4]. DF in adults is commonly found in the abdominal cavity. However, in children, they usually occur within the striated muscles of the head and neck or extremities [4, 7]. The most common reported site in the head and neck is the mandible, followed by submandibular area, neck and tongue [8]. DF has been linked to Familial Adenomatous Polyposis and Gardener syndrome with a relative risk of 850 times higher than normal [6, 7]. Staining for nuclear beta-catenin in fibromatosis has been found as high as 80–100% of cases [9] and separates deep fibromatosis from other entities in the differential diagnosis [10, 11]. Classically, histological examination shows long sweeping fascicles with elongated, slender, spindled uniform cells with pale cytoplasm set in a collagenous/myxoid stroma with no nuclear hyperchromasia, minimal cytologic atypia and variable mitotic rate. Thin walled and prominent blood vessels with perivascular edema are usually seen. In regards to management approaches, data comparing surgical and medical management remains lacking [4]. However, surgical excision remains the most common modality of choice [5]. The role of margin status is still unclear [6]. However, R0 (microscopic clearance of disease) remains preferable to R1 (macroscopic clearance but microscopic residual) and R2 (macroscopic residual) resections. The largest series by Meazza *et al.* [12] studying desmoid tumors reviewed 94 cases and concluded that recurrence was 22% in R0 and 47% in both R1 and R2. When a complete resection is not possible, conservative debulking surgery should be considered [9]. This can be especially applied in tumors of the head and neck, which pose challenges due to close proximity to vital structures as well as potential disfigurement with complete resection [6, 4]. In our case, complete resection was not possible due to the mass adhering to the skull base and other vital structures. Therefore, conservative debulking surgery was the proposed method. Chemotherapy has been suggested as both a singular and combined modality of treatment. However, it was noted that chemotherapy has a higher response as a secondary or tertiary treatment [7]. Common regimens include methotrexate and/or vinblastine, and doxorubicin with or without dacarbazine [13]. In retrospective case series of 16 patients, 13 out of 16 managed initially with surgery; however, 10 cases had local recurrence where they underwent treatment with methotrexate-vinblastine and only 2 cases had complete response [14]. Some also suggests Tamoxifen as a possible promising treatment modality for desmoid fibromatosis. This is due to suggestions of sex hormone dependency after observing tumor behavior in adult during pregnancy, menopause, contraception and tamoxifen [5, 6]. Limited data are present regarding effectiveness of radiotherapy due to concerns of long-term side effects in pediatric populations. Radiotherapy may be delivered as high-dose brachytherapy, hyperfractionated stereotactic radiation therapy or intraoperative radiation therapy. Literature suggests reserving this modality for inoperable tumors or tumors unresponsive to surgical and systemic treatments [7]. Nevertheless, a large retrospective, multicenter cohort study done in France, found that initial wait and see strategy did not have any difference in comparison to patients who underwent more aggressive initial therapies such as surgery or chemotherapy, showing that this conservative strategy does not compromise the long-term outcome [15].

## CONCLUSION

DF is a benign tumor but locally aggressive, with rapid growth and a tendency for local recurrence. Detecting the tumor before progression to a larger size will facilitate an easier surgical excision with increased likelihood of achieving microscopically negative margins. Surgical excision remains the preferred approach. However, more data are required in order to formulate an algorithm to approach and manage such tumor.
